# Problematic Internet use and life satisfaction: a moderated mediation model with social appearance anxiety as mediator and impulsivity as moderator

**DOI:** 10.3389/fpsyg.2026.1755556

**Published:** 2026-03-25

**Authors:** Ercan Aras

**Affiliations:** Vocational School of Health Services, University of Igdir, Igdir, Türkiye

**Keywords:** impulsivity, life satisfaction, moderated mediation analyses, problematic internet use, social appearance anxiety

## Abstract

Social appearance anxiety (SAA) has been recognized as a key psychological factor that negatively impact life satisfaction. Although problematic internet use (PIU) is known to be associated with increased social appearance anxiety, the mediating and moderating mechanisms linking these variables remain insufficiently explored. The current research explores how social appearance anxiety mediates and how impulsivity moderates the association between PIU and life satisfaction. The sample consists of 437 university students between the ages of 17–25 (*M* = 21.53). Data were collected using the Barratt Impulsiveness Scale-Short Form, the Problematic Internet Use Questionnaire-Short Form, the Social Appearance Anxiety Scale, and the Satisfaction with Life Scale. Pearson correlations and moderated mediation analyses were conducted using PROCESS (Model 14) macro for SPSS. Problematic internet use was found to be positively related to SAA and negatively associated to life satisfaction. SAA was statistically associated with the relationship between PIU and life satisfaction. Moreover, impulsivity moderates the indirect effect, such that individuals with low impulsivity show a stronger negative association between SAA and life satisfaction. This study highlights the complex associations among PIU, SAA, and impulsivity in relation to life satisfaction. Interventions targeting self-regulation and impulsivity may be relevant, although statistically associated conclusions cannot be drawn from the present findings.

## Introduction

### PIU and life satisfaction

PIU is characterized by a person's difficulty with self-regulation, accompanied by feelings of anxiety, anger, and tension when they have no internet access (Mittal et al., 2007). The increasing spread of digitalization has considerably increased the influence of the internet on individuals' daily lives. University students in particular often rely on the internet to fulfill both academic and social needs. In some cases, however, excessive internet use has been associated with disruption in functioning and self-control. This condition, commonly referred to in the literature as PIU, has been shown to negatively associated to life satisfaction (Kuss and Griffiths, 2015). As university students develop, they face important life tasks such as identity formation, academic achievement, and building meaningful social relationships. However, PIU may be related to difficulties in the successful completion of these developmental tasks. Thus, PIU has been identified as a factor associated with poorer social and academic skills, time management difficulties, and lower life satisfaction (Liu et al., 2022).

Cross-sectional studies indicate that university students exhibiting elevated levels of problematic internet use are more likely to experience reduced subjective wellbeing and diminished life satisfaction. As the severity of PIU increases, symptoms of depression, anxiety, and loneliness increase while positive psychological indicators such as self-esteem and life satisfaction decrease significantly (Cai et al., 2023). PIU has also been found to be associated with burnout, which in turn is related tolower life satisfaction. In addition, PIU has been related to poorer sleep behavior and lower overall quality of life (Ferrari Junior et al., 2024; Mao et al., 2024).

Life satisfaction refers to a person's overall satisfaction or dissatisfaction with their entire life as opposed to a temporary state (Vittersø et al., 2009). In other words, life satisfaction is defined as the degree to which someone feels satisfied with their life as a whole (Diener et al., 1985). A decline in life satisfaction has been associated with deteriorated academic performance and weakened relationships with others (Wang et al., 2008). Similarly, Yao and Zhong (2014) reported higher unhealthy internet use to lead to increased feelings of loneliness and lower life satisfaction. Loneliness, academic failure, loss of self-esteem, and social isolation that have related to higher levels PIU can have a negative effect on university students' life satisfaction. Therefore, PIU in university students is hypothesized to have a significant negative associated with life satisfaction, which may be related to self-regulation. Uncontrolled digital habits such as PIU are thought to be related to depleted self-regulatory capacities (LaRose et al., 2003). In particular, PIU may impair, or may be associated with impairments in self-regulatory mechanisms through processes such as distractibility, time management difficulties, procrastination, and excessive reward behavior. Over time, these impairments may be related to loneliness, poor academic performance, and low self-esteem, and may be linked to lower life satisfaction (Wills et al., 2011). In this context, the association between PIU and lower life satisfaction may be interpreted within the framework of self-regulation deficits. Accordingly, the following hypothesis has been formulated:

H1: *PIU is negatively associated with life satisfaction*.

### Social appearance anxiety as a mediator

SAA refers to a person's concern about how others evaluate their physical appearance (Hart et al., 2008a). This anxiety is not limited to physical appearance but also relates to social acceptance, perceived self-worth, and social comparisons. Particularly among young adults in the university student group, concern about social appearance tends to increase and become more pronounced. In addition to the increase in PIU, an increase in SAA has also been observed (Hou et al., 2019). People who spend long periods of time in digital environments are often exposed to idealized body images, which is associated with negative self-evaluation and increased anxiety about their own appearance (Fardouly et al., 2015). Consequently, a significant positive relationship has been observed between PIU and SAA (Marino et al., 2018). This association may be particularly evident in young adults such as university students, who are concerned about their social appearance. In addition, SAA has been reported to have a negative relationship with life satisfaction. People who fear their appearance being negative evaluated often report experience social isolation, low self-esteem, and reduced self-actualization, which contributes to lower life satisfaction (Sandoz et al., 2013). Studies have shown SAA to be associated with depression and self-confidence. When SAA increases, depressive symptoms increase and life satisfaction decreases (Ryding et al., 2024). Therefore, SAA may serve as a mediating risk factor in the relationship between PIU and life satisfaction. This mediating process may serve as a critical link in the pathway between people's digital behaviors and overall psychological wellbeing.

### Self-regulation theory and the moderating role of impulsivity

Self-regulation theory states that behavior is influenced by feedback from the environment (Hart et al., 2008a). However, people with weak self-regulation skills are more likely to display maladaptive behaviors and report reduced wellbeing when confronted with challenges. Kardefelt-Winther (2017) surmised PIU may serve as a coping mechanism for those with inadequate self-regulation. This pattern may be associated with higher SAA, as individuals who do not receive social approval become more concerned about their physical appearance (Hart et al., 2008b). Consistent with this view, higher PIU has been found to be associated with higher SAA, and higher SAA with lower life satisfaction (Fardouly et al., 2015). Self-regulation theory is based on individuals' capacity to control their behavior in line with their goals, direct their attention, and manage their impulses. Problematic internet use (PIU) is directly related to the weakening of this capacity. PIU leads to impairments in time management and attention control, thereby weakening self-regulation mechanisms. This weakness increases individuals' social appearance anxiety (SAA), since those with low self-regulation capacity are more likely to seek social approval in online environments. Increased SAA, in turn, reduces life satisfaction. Thus, the chain PIU → weakened self-regulation → increased SAA → decreased life satisfaction can be explained within the framework of self-regulation theory. This approach is consistent with recent studies emphasizing the critical role of self-regulation mechanisms in digital media use (Rebello et al., 2024; Hrbackova et al., 2025). Moreover, Billieux and Van der Linden (2013) argued that problematic internet use is closely related to self-regulation deficits and that these deficits negatively affect anxiety and life satisfaction. Importantly, impulsivity appears to play a moderating role in these associations. Individuals with high impulsivity often struggle to regulate their emotions and behaviors, which exacerbates the negative association between SAA and life satisfaction (Whiteside and Lynam, 2001). From the perspective of self-regulation theory, impulsivity is conceptually linked to lower self-regulatory capacity, thus reducing their ability to cope with SAA. Self-regulation theory provides a conceptual lens for understanding the relationships among impulsivity, problematic internet use, social appearance anxiety, and life satisfaction. Although core components of self-regulation (e.g., goal monitoring, executive control, regulatory capacity) were not directly measured in this study, impulsivity can be conceptualized as a vulnerability in self-regulatory functioning, and problematic internet use may reflect difficulties in regulating online behaviors. Thus, the proposed model is theoretically informed by self-regulation perspectives, even though it does not directly operationalize all components of the theory. In the present model, SAA is hypothesized to statistically mediate the association between PIU and life satisfaction, with impulsivity moderating the association between PIU and life satisfaction. In other words, higher of SAA and impulsivity are expected to be related to lower life satisfaction. Unlimited internet access, social media's characteristic of immediate feedback, and being susceptible to social comparison may particularly intensify problematic online behaviors in individuals with high SAA levels, which is, in turn, linked to lower life satisfaction. Although previous studies have focused on different variables related to life satisfaction and PIU, few have examined both impulsivity and SAA together in a Turkish sample. Therefore, the following hypotheses have been formulated:

*H2: PIU is positively associated with SAA*.
*H3:* SAA is negatively associated with life satisfaction.
*H4: SAA statistically mediates the association between PIU and life satisfaction*.

### Impulsivity as a moderating variable

Impulsivity is a complex, multifaceted trait that denotes an individual's propensity to engage in actions without prior reflection or evaluation of potential outcomes, typically manifesting as rapid and spontaneous responses. This trait is commonly associated with deficits in behavioral control and is often linked to impatience, lack of planning, and distractibility. Research has shown higher impulsivity to be negatively associated with life satisfaction and overall quality of life (Sánchez-Sánchez et al., 2023). People with high impulsivity tend to postpone long-term goals, make risky decisions, and have problems with emotion regulation (Whiteside and Lynam, 2001). In particular, studies examining the associations of impulsivity on life satisfaction have suggested emotional and cognitive impulsivity may hinder one's ability to lead a fulfilling life (Xie et al., 2025). These findings indicate impulsivity not only a behavioral factor but also an important variable associated with overall life satisfaction. Such characteristics are consistent with disruptions in self-regulatory processes, which in turn tend to be linked with negatively impact life satisfaction.

Impulsivity has been repeatedly associated with impairments in the self-regulatory mechanisms that hinder goal-directed behavior and emotional regulation. The theory of self-regulation is based on one's ability to plan, monitor, and control goal-oriented behaviors (Baumeister and Vohs, 2007). In this framework, impulsivity is associated with one's underutilization of these self-regulatory mechanisms. Impulsive people often have difficulty pursuing long-term goals and tend to seek immediate gratification, which can hinder a planned, fulfilled life. From the perspective of self-regulation theory, impulsivity is a factor that may negatively impact one's inner balance and goal-oriented behaviors. Accordingly, impulsivity may serve as a moderator in the relationship between such psychosocial variables as social performance anxiety and life satisfaction. Recent studies have shown impulsivity to play a moderating role in the relationships among social anxiety, PIU, loneliness, and life satisfaction (Miao et al., 2024). In line with self-regulation theory, impulsivity can therefore be seen as a critical personality trait is associated with that contributes to lower life satisfaction due to one's failure to effectively regulate their behaviors and emotional responses. Investigating the moderating effect of impulsivity on the relationship between SAA and life satisfaction may provide valuable theoretical and practical insights. In this context, the following hypothesis has been formulated:

H5: *Impulsivity moderates the association between PIU and life satisfaction through SAA*.

### The present study

**Figure 1:**
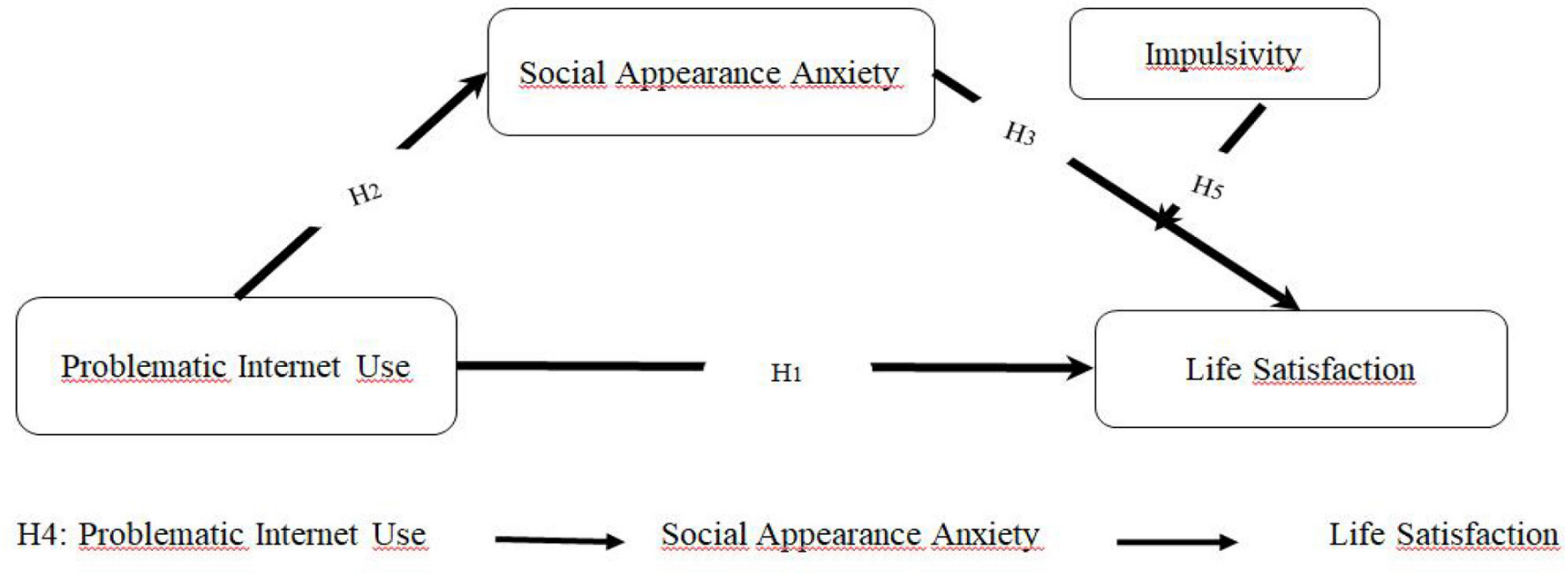
The hypothesized model.

While previous research has identified a link between problematic internet use and life satisfaction, information regarding the associations involving SAA and impulsivity remains limited. This study aims to examine how SAA is statistically associated with the relationship among impulsivity, PIU, and life satisfaction. The research employs a moderated mediation model based on self-regulation theory to investigate the associations among these variables. According to self-regulation theory, individuals with low self-regulatory capacity may be more likely to report increased internet use and heightened SAA, which may be associated with lower life satisfaction. Additionally, impulsivity tends to be higher among individuals with poor self-regulation. Furthermore, impulsivity is examined as a potential moderator in the association between social appearance anxiety and life satisfaction. Accordingly, the study hypothesizes that PIU is negatively associated with life satisfaction (H1) and positively associated with SAA (H2). SAA is further expected to be negatively associated with life satisfaction (H3). In addition, SAA is hypothesized to statistically mediate the relationship between PIU and life satisfaction (H4). Finally, impulsivity is tested as a moderator of the association between SAA and life satisfaction (H5). The proposed model is illustrated in Figure 1.

## Method

### Participants

This study was conducted at a university located in Türkiye's Eastern Anatolia Region. The sample consists of 437 undergraduate students, including 318 females (72.77%) and 119 males (27.23%). The participants have a mean age of 21.53 years. In terms of the participants' daily internet usage, 38% (*n* = 166) reported using the internet for 0–2 h, 33.6% (*n* = 147) for 2–4 h, 17.4% (*n* = 76) for 4–6 h, and 12% (*n* = 48) for 6–8 h. Regarding the participants' perceived socioeconomic status, 69.79% (*n* = 305) identified as middle level, 20.59% (*n* = 90) as low level, and 9.62% (*n* = 42) as high level (Table 1).

**Table 1:** Participant demographics.

**Variables**		***f***	**%**	***M***	***SD***
**Gender**					
	Female	318	72.77		
	Male	119	27.23		
Age				21.53	4.31
**Perceived Income Level**					
	Low	90	20.59		
	Middle	305	69.79		
	High	42	9.62		
**Internet use duration**					
	0–2 h	166	38		
	2–4 h	147	33.6		
	4–6 h	76	17.4		
	6–8 h	48	11		

f, frequency; %, percentage; M, mean; SD, standard deviation.

### Procedure

Ethical approval for the present study was obtained from the Ethics Committee of Igdir University in Türkiye. Prior to completing the survey, participants received detailed information regarding the study's objectives and procedures, and their informed consent was obtained, with particular emphasis on the voluntary nature of their participation. The data were collected between February and May, 2025. A total of 437 participants completed the study, indicating an adequate sample size for the analyses.

### Data collection instruments

*Barratt Impulsiveness Scale-Short Form* (BIS-15) Patton et al. (1995) was adapted to Turkish by Tamam et al. (2013). This scale is a four-point Likert-type instrument consisting of 15 items (sample items: “I act on the spur of the moment” and “I do things without thinking”). The first five items are reverse scored, and total scores can range from 15 to 60, with higher scores indicating greater impulsivity levels. The scale includes three subscales: non-planning, attentional impulsivity, and motor impulsivity. In the original adaptation study, Cronbach's alpha coefficients were reported as 0.82 for the overall scale and 0.80, 0.64, and 0.70 for the respective subscales. In the present study, the internal consistency of the scale was similarly confirmed, with a Cronbach's alpha of 0.82. In the conducted confirmatory factor analysis (CFA), the model's fit indices were examined, and χ^2^/*df* = 3.15, RMSEA = 0.07, SRMR = 0.04, CFI = 0.94, GFI = 0.96, and AGFI = 0.92 were calculated. These findings indicate that the model demonstrated good fit.

*Problematic Internet Use Questionnaire*- *Short Form* was originally developed by Demetrovics et al. (2016) and adapted to Turkish by Göktaş et al. (2018). The scale is a five-point Likert-type. Sample items include “How often do you fail to reduce the time you spend online, even though you want to?” and “How often do you try to conceal the amount of time you spend on the internet?” Total scores can ranges from 6 to 30. The scale includes three subdimensions: obsession, neglect, and control disorder, elevated scores reflect higher levels of problematic internet use. The current study has calculated a Cronbach's alpha of 0.85. In the conducted confirmatory factor analysis (CFA), the model's fit indices were examined, and χ^2^/*df* = 4.12, RMSEA = 0.08, SRMR = 0.03, CFI = 0.99, GFI = 0.98, and AGFI = 0.94 were calculated. These findings indicate that the model demonstrated good fit.

*Satisfaction With Life Scale* was developed by Diener et al. (1985) and adapted to Turkish by Dagli and Baysal (2016). The instrument includes five statements, which respondents rate on a five-point Likert scale. Sample items include “I have a life close to my ideals” and “My living conditions are excellent.” The original scale reported an internal consistency of 0.88 and test-retest reliability of 0.97. In the present study, Cronbach's alpha has been determined to be 0.87. Elevated scores on the scale correspond to higher levels of life satisfaction. In the conducted confirmatory factor analysis (CFA), the model's fit indices were examined, and χ^2^/*df* = 1.17, NFI = 0.99, NNFI = 1.00, CFI = 1.00, GFI = 0.99, AGFI = 0.97, RMSEA = 0.030, and SRMR = 0.019 were calculated. These findings indicate that the one-factor structure of the scale showed good fit according to the confirmatory factor analysis. These findings indicate that the model demonstrated good fit.

*Social Appearance Anxiety Scale* was developed by Hart et al. (2008a) and adapted to Turkish by Dogan (2010). Higher scores on the scale signify greater levels of SAA. The scale consists of 16 items and one dimension. The first item is reverse scored. Sample items include “I feel comfortable about how I look” and “I worry that people will not find me attractive.” The original scale demonstrated an internal consistency coefficient of 0.91 and a test-retest reliability of 0.80. In the present study, Cronbach's alpha was calculated as 0.95. In the conducted confirmatory factor analysis (CFA), the model's fit indices were examined, and χ^2^ = 143.79 (*N* = 254, *p* = 0.01), RMSEA = 0.051, NFI = 0.98, CFI = 0.99, IFI = 0.99, RFI = 0.98, GFI = 0.93, and AGFI = 0.90 were calculated. These findings indicate that the model demonstrated good fit.

### Statistical analysis

The research data were processed and analyzed utilizing IBM SPSS software (ver. 27) and Hayes' PROCESS Macro version 4.2. The assumptions of normality were tested first, and all values were found to lie within the ±1.5 range, supporting the assumption of normality in the data. Prior to evaluating multicollinearity, Pearson correlation analysis was conducted to investigate the relationships among the variables. According to Hair et al. (2019), tolerance values > 0.10, variance inflation factor (VIF) values < 10, and condition indices < 30 indicate multicollinearity to not be present. Based on these criteria, no multicollinearity problems were identified in the dataset. Next, the analysis examined the moderating role of impulsivity in the relationship between SAA and life satisfaction, conducting a bootstrap analysis to determine the significance of the path coefficients (Hayes, 2018). Within this framework, SAA was treated as a mediator variable, while impulsivity was considered a moderator variable. Specifically, SAA was modeled as a mediator in the relationship between PIU and life satisfaction, with impulsivity serving as a moderator. The bootstrap method employed a 95% confidence interval and 10,000 resampling iterations. The analyses were conducted using PROCESS Macro Model 14. All constructs were treated as observed composite variables calculated as mean scores of their respective validated scales. This decision was based on the strong internal consistency coefficients observed in the present sample and on the primary aim of the study, which was to examine conditional process relationships rather than to test a full measurement model. This model was selected because it allows testing moderation specifically on the mediator-to-outcome path, which aligns with our theoretical framework derived from self-regulation theory. In particular, impulsivity was hypothesized to weaken the effect of social appearance anxiety on life satisfaction, making Model 14 the most appropriate specification for the present study.

## Results

As shown in Table 2, the skewness and kurtosis values indicate that the data are normally distributed. It is emphasized that skewness and kurtosis values should fall between −1.5 and +1.5 to be considered appropriate for normal distribution (Garson, 2012). Examination of the correlation coefficients revealed significant relationships among the variables (*p* < 0.05). Accordingly, the variables were found to be correlated with each other, with correlation coefficients ranging from r = −0.31 to 0.44.

**Table 2:** Descriptive statistics and correlations.

	**Descriptive statistics**				**Correlations**			
**Variables**	***M***	***SD***	**Skewness**	**Kurtosis**	α	**1**	**2**	**3**
1. PIU	13.43	5.248	0.661	0.003	0.85	–		
2. Impulsivity	36.11	4.842	0.728	0.770	0.82	0.32^**^	–	
3. SAA	32.17	13.052	0.963	0.173	0.95	0.44^**^	0.33^**^	–
4. Life satisfaction	14.05	4.618	−0.122	−0.930	0.87	−0.22^**^	−0.14^**^	−0.31^**^

PIU, Problematic Internet Use; SAA, Social Appearance Anxiety; M, Mean; SD, Standard Deviation.

^**^indicates statistical significance at *p* < 0.01.

### Moderated mediating analysis

After identifying significant relationships among the variables, the study examined SAA's mediating role and impulsivity's moderating role in the relationship between PIU and life satisfaction. Table 3 presents the findings from the mediation and moderation analyses.

**Table 3:** Moderated mediational analysis.

**Variables**	**M (SAA)**						**Y (Life satisfaction)**					
	**β**	***SE***	***t***	***p***	**LLCI**	**ULCI**	**β**	***SE***	***t***	***p***	**LLCI**	**ULCI**
X (PIU)	1.09	0.11	9.90	< 0.01	0.88	1.31	−0.07	0.46	−0.15	< 0.01	−0.16	0.02
C (gender)	0.78	1.24	0.63	>0.05	−1.65	3.21	−0.11	0.02	−5.64	>0.05	−0.14	−0.07
C (age)	−0.07	0.13	−0.52	>0.05	−0.34	0.19	−0.05	0.05	−1.15	>0.05	−0.15	0.04
M(SAA)							0.15	0.03	5.82	< 0.01	−0.20	−0.10
W (Impulsivity)							−0.11	0.02	−5.64	< 0.05	−0.14	−0.07
M^*^W (SAA ^*^ Impulsivity)							−0.06	0.02	−3.01	< 0.05	−0.10	−0.02
	*R* = 0.36, *R*^2^ = 0.13						*R* = 0.14, *R*^2^ = 0.02					
	*F*_(6, 430)_ = 10.89, *p* < 0.001						*F*_(1, 430)_ = 10.29, *p* < 0.001					

X, Independent variable; PIU, Problematic Internet Use; M, Mediator; SAA, Social Appearance Anxiety; W, Moderator; C, Control variable.

As presented in Table 3, the direct effect of PIU on SAA is statistically significant (β = 1.09, 95% CI [0.88, 1.31], *p* < 0.001). However, the covariates of gender (β = 0.78, 95% CI [−1.65, −3.21], *p* > 0.05) and age (β = −0.07, 95% CI [−0.34, −0.19], *p* > 0.05) had no significant effect on SAA. These variables collectively explain 13% of the variance in SAA, which is statistically significant [*F*_(6, 430)_ = 10.89, *p* < 0.001]. Subsequently, the study investigated the moderating role of impulsivity in the relationship between SAA and life satisfaction. The direct effect of PIU on life satisfaction is found to be significant but negative (β = −0.07, 95% CI [−0.16, −0.02], *p* < 0.001). In addition, the effect of SAA on life satisfaction is also significant (β = −0.15, 95% CI [0.20, −0.10], *p* < 0.001), indicating that higher levels of SAA are associated with lower levels of life satisfaction. Impulsivity demonstrates a significant negative effect on life satisfaction (β = −0.11, 95% CI [−0.14, −0.07], *p* < 0.05). Moreover, the interaction between SAA and impulsivity was found to be statistically significant (β = −0.06, 95% CI [−0.10, −0.02], *p* < 0.05), suggesting that impulsivity moderates the relationship between SAA and life satisfaction. However, with gender as a control variable (β = −0.11, *p* > 0.05), and age (β = −0.05, *p* > 0.05) are not seen to significantly predict life satisfaction. The overall model explained a small proportion of variance in life satisfaction (*R*^2^ = 0.02), indicating that although statistically significant, the practical effect size was modest. The inclusion of the interaction term (SAA × Impulsivity) contributed a small but statistically significant increment in explained variance, a finding that is statistically significant [*F*_(1, 430)_ = 10.29, *p* < 0.001], thus supporting Hypothesis 3.

This study further investigates the moderating role of impulsivity in the relationship between and life satisfaction using Model 14 of the PROCESS Macro. The findings are presented in Table 4.

**Table 4:** Conditional indirect effects of PIU on life satisfaction.

**Impulsivity**	**Indirect effect**	**Boot SE**	**Boot LLCI**	**Boot ULCI**
−1 SD	−0.16	0.04	−0.23	−0.10
M	−0.12	0.03	−0.17	−0.07
+1 SD	−0.07	0.02	−0.12	−0.02
**Index of moderated mediation**				
Impulsivity	0.01	0.03	0.03	0.02

Table 4 presents the conditional indirect effects of PIU on life satisfaction through SAA at three levels of impulsivity (−1 SD, mean, +1 SD). The indirect effect was significant at all levels, with the strongest effect observed at low impulsivity. Moreover, the index of moderated mediation was significant (β = 0.01, 95% CI [0.02, 0.03]), indicating that impulsivity significantly moderates the indirect pathway from PIU to life satisfaction via SAA. These findings suggest that impulsivity plays a meaningful role in shaping the mediating effect of social appearance anxiety in this relationship.

**Figure 2:**
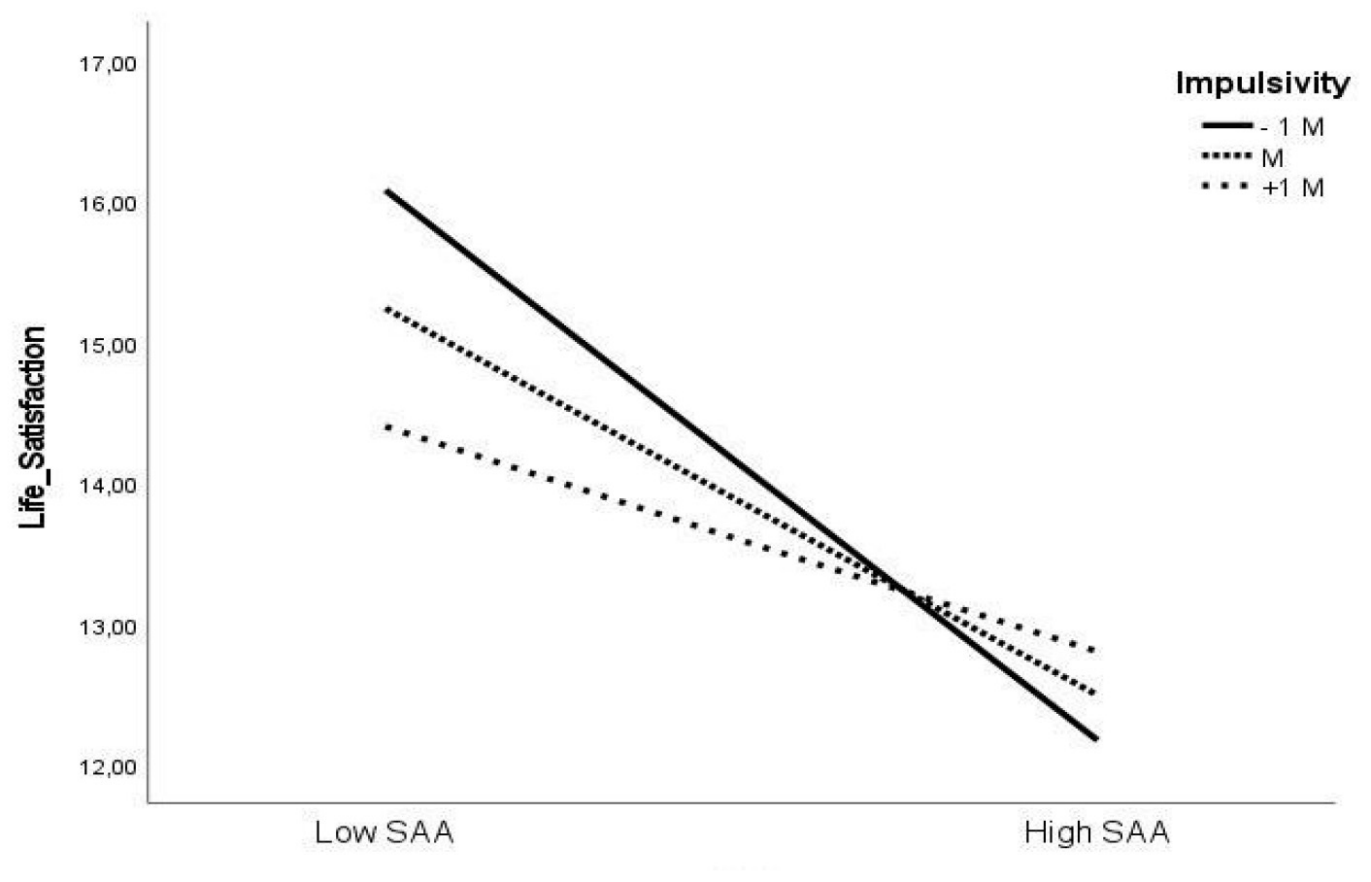
Interaction plot illustrating the moderating effect of impulsivity on the relationship between social appearance anxiety and life satisfaction.

Figure 2 illustrates the conditional indirect effects of PIU on life satisfaction through SAA at low, mean, and high levels of impulsivity. The strength of the indirect effect decreases as impulsivity increases.

The study examined the conditional indirect effect of SAA in the relationship between PIU and life satisfaction across three levels of impulsivity: low (−1 SD), mean, and high (+1 SD). The results demonstrate the indirect effect of PIU on life satisfaction through SAA to be statistically significant at all impulsivity levels. Specifically, at low impulsivity (−1 SD), the effect was strongest (β = −0.1639, 95% CI [−0.2344, −0.1007], SE = 0.0344). At the mean level of impulsivity, the effect remained significant though slightly reduced (β = −0.1156, 95% CI [−0.1669, −0.0710], SE = 0.0246), while at high impulsivity (+1 SD), the indirect effect further weakened but remained significant (β = −0.0673, 95% CI [−0.1192, −0.0223], SE = 0.0247).

As visualized in Figure 2, higher impulsivity levels are associated with a weaker negative association between SAA and life satisfaction. When impulsivity is low, individuals with higher levels of SAA report substantially lower life satisfaction. At average impulsivity, this negative association persists but is less pronounced. At high impulsivity, SAA continues to negatively predict life satisfaction, yet the strength of this association diminishes notably. Impulsivity significantly moderated the relationship between social appearance anxiety and life satisfaction, and the index of moderated mediation was statistically significant. This finding indicates that impulsivity meaningfully shapes the indirect pathway from problematic internet use to life satisfaction via social appearance anxiety, with the strongest negative effect observed at lower impulsivity levels.

## Discussion

The findings of this study indicate that SAA statistically mediates the association between PIU and life satisfaction. In addition, impulsivity was found to moderate the relationship between SAA and life satisfaction. Overall, the results are consistent with the proposed model at the level of statistical associations. Although self-regulation theory informed the conceptual development of the model, core self-regulatory processes were not directly assessed. Accordingly, the theoretical integration should be understood as conceptual rather than as direct empirical verification of specific self-regulation mechanisms. Future studies including explicit measures of regulatory capacity would provide a more rigorous test of the proposed framework.

The results suggest that higher PIU levels are statistically associated with lower life satisfaction, with SAA functioning as an intermediary variable in this association. Longitudinal research also shows that life satisfaction can mediate long-term outcomes of problematic internet use, reinforcing the importance of subjective wellbeing in internet addiction models (Shi et al., 2025) These findings align with prior research reporting negative associations between excessive internet use and wellbeing (Kuss and Griffiths, 2015; Kardefelt-Winther, 2017). Excessive internet engagement has been linked to social withdrawal and reduced satisfaction in offline life domains. From a self-regulatory perspective, problematic internet use may reflect difficulties in managing coping strategies, which in turn are associated with somewhat lower levels of life satisfaction. Recent findings also indicate that problematic internet use predicts diminished life satisfaction through psychological distress (Codina et al., 2025), which conceptually aligns with our results showing SAA as a pathway to reduced wellbeing. Similarly, PIU was found to be positively associated with SAA. This pattern is consistent with research suggesting that repeated exposure to appearance-based social comparison in digital environments is linked to heightened appearance-related concerns (Fardouly et al., 2015; Ding et al., 2023). Platforms emphasizing visual self-presentation may increase awareness of physical appearance and social evaluation. Within broader motivational frameworks, such as self-determination theory (Deci and Ryan, 2000), appearance-based comparison processes may be linked to greater reliance on external validation, which is associated with higher appearance-related anxiety. SAA was also negatively associated with life satisfaction. This finding corresponds with prior studies indicating that heightened appearance-related concerns are related to lower self-esteem and diminished wellbeing (Tylka and Wood-Barcalow, 2015). Individuals experiencing elevated SAA may engage in persistent self-evaluative comparisons, which are statistically linked to somewhat lower life satisfaction. The mediation analysis further indicated that SAA statistically accounts for part of the association between PIU and life satisfaction. This suggests that appearance-related anxiety may represent one pathway through which problematic internet use is associated with lower wellbeing. However, given the cross-sectional design and the modest proportion of variance explained in life satisfaction, these findings should be interpreted as associative patterns rather than directional or practically large effects. The relatively small proportion of explained variance in life satisfaction suggests that this outcome is influenced by a wide range of additional psychological, social, and contextual factors beyond those included in the present model. Therefore, the findings should be interpreted as reflecting a partial explanatory framework rather than a comprehensive account of life satisfaction determinants

Finally, impulsivity was found to moderate the association between SAA and life satisfaction, such that the negative association was stronger at lower levels of impulsivity. While impulsivity significantly moderated the association between SAA and life satisfaction, the index of moderated mediation was not statistically significant. Therefore, the moderated mediation hypothesis was not fully supported. Contrary to initial theoretical expectations, higher impulsivity attenuated the strength of this association. This finding does not suggest that impulsivity functions as a protective factor; rather, it may reflect a more complex interaction pattern. Although prior studies have conceptualized impulsivity as a risk amplifier (Megías-Robles et al., 2024; Makarenko et al., 2025), these findings were largely based on direct or mediational models. By contrast, the present study examined impulsivity as a moderator, highlighting that its effects may be conditional rather than uniformly additive. Differences in outcome variables and cultural context may further explain the divergence. Thus, impulsivity's role may depend on analytical specification and contextual factors rather than representing a stable risk-enhancing trait. Although some prior studies have conceptualized impulsivity as a risk amplifier, recent meta-analytic evidence confirms that problematic internet use is significantly associated with impulsivity-related symptoms, suggesting a robust link between maladaptive digital engagement and reduced inhibitory control (Augner et al., 2023). One possible explanation is that individuals with higher impulsivity may already report comparatively lower baseline life satisfaction, thereby reducing the incremental association of SAA (a potential floor effect). Alternatively, impulsive tendencies may be linked to more rapid emotional disengagement from appearance-related concerns, limiting sustained cognitive elaboration. Further longitudinal and experimental research is needed to clarify the mechanisms underlying this interaction. From a self-regulation perspective, problematic internet use may reflect difficulties in behavioral monitoring and inhibitory control in digital contexts, whereas social appearance anxiety may consume limited regulatory resources through persistent self-evaluative processes. Impulsivity, characterized by reduced inhibitory control, may shape how individuals cope with appearance-related distress. Although regulatory capacity was not directly measured, the findings are conceptually consistent with broader models of self-regulatory vulnerability rather than providing direct empirical confirmation of regulatory mechanisms. Importantly, these findings should be interpreted within the sociocultural context of Turkish university students. Cultural norms related to appearance, social comparison, and digital engagement may shape the observed associations, underscoring the need for cross-cultural replication. Moreover, while the observed associations are statistically significant, the overall explanatory power of the model is modest, highlighting that life satisfaction is influenced by a wide range of psychological, social, and contextual factors beyond the variables included in the current study.

## Limitations and future directions

Although this study contributes to the literature, it has several limitations. First, the cross-sectional design precludes statistically associated inferences, and the reported mediation and moderation pathways should be interpreted as statistical associations rather than directional effects. Future longitudinal or experimental research could more rigorously examine these relationships. Second, the sample was drawn exclusively from students at a single university in Eastern Anatolia, Türkiye, limiting generalizability; sociocultural norms may shape the observed associations. Third, although age and gender were controlled, other relevant covariates—such as depressive symptoms, general anxiety, social media use intensity, and body mass index—were not assessed, leaving the possibility of omitted variable bias. Future studies should include a broader set of covariates. Fourth, all measures were collected via self-report at a single time point, which may increase susceptibility to common method bias; using multiple data sources or temporally separated measurements could mitigate this risk. Finally, while findings suggest that impulsivity may moderate the relationship between social appearance anxiety and life satisfaction, these results are preliminary and require replication in other populations and designs.

## Conclusions

This study has found impulsivity to significantly moderate the relationship between SAA and life satisfaction, with SAA mediating the relationship between problematic internet use and life satisfaction. The study makes an important contribution to understanding the relationship between PIU and life satisfaction. According to self-regulation theory, PIU is associated with SAA. The results indicate that PIU, SAA, and impulsivity are significantly associated with life satisfaction. This study identifies digital and psychological variables that are statistically associated with life satisfaction, thus providing a basis for preventative interventions by psychological counselors and educators. Furthermore, the study has shed light on the indirect effects of young adults' digital behavior on their psychological wellbeing.

## Data Availability

The raw data supporting the conclusions of this article will be made available by the authors, without undue reservation.
